# Evaluation of Small Vessel Bifurcation Stenting Using the Double‐Kissing Culotte and Culotte Technique in Acute Coronary Syndrome: 12‐Month Clinical Outcomes

**DOI:** 10.1002/clc.70043

**Published:** 2024-11-15

**Authors:** Mateusz Barycki, Piotr Rola, Adrian Włodarczak, Szymon Włodarczak, Maciej Pęcherzewski, Piotr Włodarczak, Artur Jastrzębski, Łukasz Furtan, Andrzej Giniewicz, Adrian Doroszko, Maciej Lesiak

**Affiliations:** ^1^ Department of Cardiology Provincial Specialized Hospital Legnica Poland; ^2^ Faculty of Medicine Wroclaw University of Science and Technology Wroclaw Poland; ^3^ Department of Cardiology The Copper Health Centre (MCZ) Lubin Poland; ^4^ Faculty of Pure and Applied Mathematics Wroclaw University of Science and Technology Wroclaw Poland; ^5^ Department of Cardiology, Center for Heart Diseases, 4th Military Hospital, Faculty of Medicine Wroclaw University of Science and Technology Wroclaw Poland; ^6^ 1st Department of Cardiology University of Medical Sciences Poznan Poland

**Keywords:** acute coronary syndrome, endovascular procedures, percutaneous coronary intervention

## Abstract

**Introduction:**

Patients with small vessels who undergo percutaneous coronary intervention (PCI) with subsequent multiple implantation of drug‐eluting stents remain at a higher risk of unfavorable outcomes. In complex cases where maintaining flow to all side branches is part of contemporary practice, using two‐stent techniques may be appropriate. This study aims to evaluate the efficacy of double‐kissing (DK) culotte technique in comparison to culotte technique in the context of small‐vessel therapy in patients with acute coronary syndrome (ACS).

**Methods:**

This substudy of the Lower Silesia culotte Bifurcation Registry retrospectively analyzed patients who underwent ACS‐PCI using DK culotte or culotte technique for bifurcation lesions in small vessels, defined as having at least one branch with a diameter of 2.75 mm or less. The primary endpoint was target lesion failure (TLF), a composite of cardiovascular death, target vessel myocardial infarction, or clinically driven target lesion revascularization (TLR) at 1‐year follow‐up. The secondary endpoint included major adverse cardiac events (MACE).

**Results:**

The DK culotte group (*n* = 49) and the culotte group (*n* = 52) were compared, with 12‐month follow‐up showing lower TLF in the DK culotte group (8.2% vs. 19.2%, *p* = 0.082). Similar results were observed for TLR (6.1% vs. 13.5%; *p* = 0.161), stent restenosis (4.1% vs. 9.6%; *p* = 0.203), and MACE (18.4% vs. 25%; *p* = 0.344).

**Conclusion:**

For bifurcation lesions with a small‐diameter artery, the DK culotte technique may reduce TLF and MACE compared to the culotte technique. However, given the limited sample size and the absence of statistical significance, these findings remain preliminary and require further investigation.

## Graphical abstract

Limited data exist on coronary bifurcation outcomes using the DK‐culotte technique. A 1‐year follow‐up of ACS patients with PCI of bifurcation lesions with small vessels (one vessel ≤ 2.75 mm) compared DK‐culotte (*n* = 49) versus culotte (*n* = 52). Results showed TLF: 8.2% versus 19.2% (*p* = 0.086) and MACE: 18.4% versus 25% (*p* = 0.344).

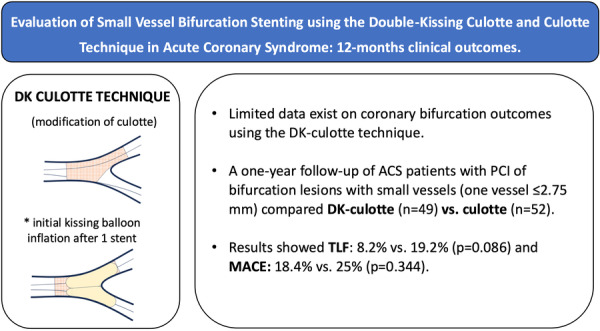

## 1 Introduction

True coronary bifurcation disease doesn't often occur in patients undergoing percutaneous coronary intervention (PCI) [1], but it remains a relevant clinical issue. Patients with small vessels who receive PCI with subsequent implantation of drug eluting stent (DES) are at higher risk of unfavorable outcomes. The postsmall vessel PCI subpopulation is more prone to suffer from target lesion revascularization (TLR) episodes, primarily due to higher rates of restenosis or stent thrombosis [2, 3]. Revascularization guidelines support the provisional stenting technique as more efficient for the majority of bifurcation lesions. However, in the cases of high complexity, particularly in the acute coronary syndrome (ACS) subset, where preservation of accurate flow to all side branches is critical, two‐stenting techniques may be appropriate. Several different stenting techniques have been used in clinical practice to date, but little is known about their safety and efficacy in small vessel disease. The culotte technique was first described by Chevalier et al. [4] in 1998 and has since become an established part of contemporary practice. Recently published data suggest that the culotte technique may be more effective than T and protrusion (TAP) stenting in the long‐term preservation of a side branch (SB) of a bifurcation [5]. Several preclinical data suggest that a variation of the classical culotte technique, the so‐called double‐kissing (DK) culotte technique, in which an initial kissing balloon dilatation is performed immediately after SB stenting and before main branch (MB) stent implantation, may facilitate the PCI procedure and provide better morphological characteristics after stenting [6, 7]. The preliminary published data on outcomes following the DK Culotte technique are promising [8, 9], however, there is a lack of data regarding its application in small vessel disease. This subanalysis of the Lower Silesia Culotte Bifurcation Registry [ClinicalTrials.gov: NCT06284057] focuses on the safety and efficacy of Culotte and DK‐Culotte techniques in a real‐world ACS subset, specifically in the context of small‐vessel therapy.

## 2 Materials and Methods

### 2.1 Study Population

**Figure 1:**
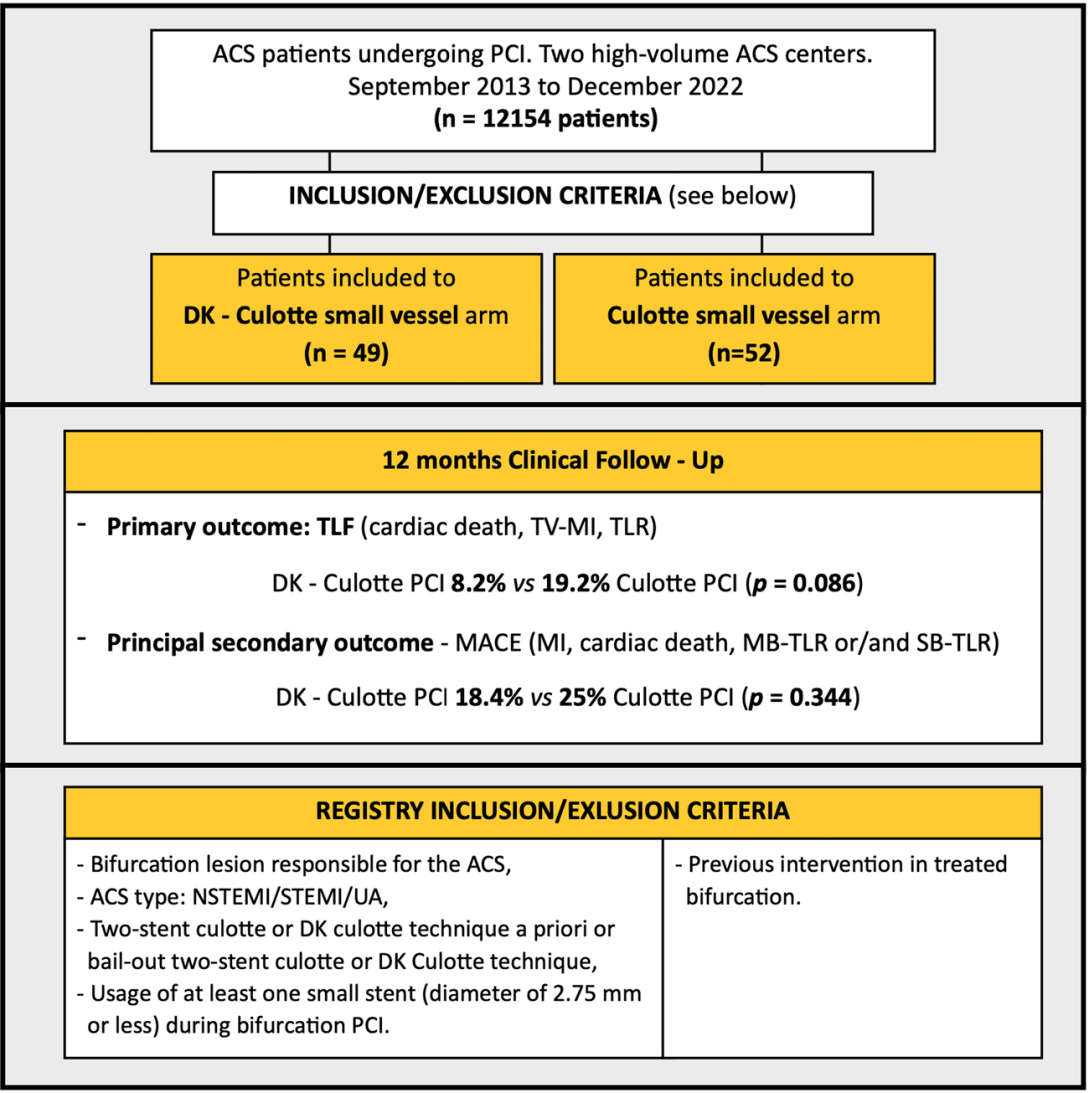
Central illustration. Evaluation of small vessel bifurcation stenting using the Double‐Kissing Culotte or Culotte Technique in Acute Coronary Syndrome. Twelve months clinical outcomes. ACS, acute coronary syndrome; DK culotte, double‐kissing culotte; MB, main branch; MI, myocardial infarction; PCI, percutaneous coronary intervention; SB, side branch; TLF, target lesion failure; TLR, target lesion revascularization; TV, target vessel.

This is a subanalysis of the retrospective Lower Silesia Culotte Bifurcation Registry (ClinicalTrials.gov: NCT06284057). The substudy population consists of consecutive subjects who underwent PCI for ACS with culprit lesions located in the coronary bifurcation and received at least one small stent (diameter of 2.75 mm or less) during the DK culotte or culotte procedure between September 2013 and December 2022 (see Figure 1). The indication for PCI was based on the decision of the cardiac team, either on the basis of clinical indication (persistent ischemia, unwillingness to accept alternative treatment options, coronary artery disease suitable for primary PCI according to ESC/ESH recommendations). The decision to perform two‐stent PCI was left to the discretion of the operator. The study included only patients with de novo lesions not previously treated by PCI with both branches suitable for DES implantation (reference size at least 2 mm). There were no other clinical or vascular exclusion criteria (lesion anatomy, length, tortuosity, severity). All patients were thoroughly informed of all therapeutic options and provided the required written informed consent. The local ethics committee approved the study—Lower Silesian Medical Association (Poland)—approval number—01/BO/2023.

### 2.2 Study Endpoints

The primary endpoint was target lesion failure (TLF) defined as cardiac death, target vessel myocardial infarction (TV‐MI), or TLR. The observation period was 12 months. Other endpoints included the principal secondary outcome—major adverse coronary events (MACE) which encompassed MI, cardiac death, MB‐TLR, or/and SB‐TLR. Additional endpoints were target vessel MI, all‐cause death, stent restenosis, and thrombosis. TLR, stent thrombosis, and restenosis were defined according to the Academic Research Consortium—2 consensus [9].

### 2.3 Statistical Analysis

Statistical analyses were performed using R programming language. To compare subjects between groups, the nonparametric two‐sample Mann–Whitney *U* test was used for continuous variables and Fisher's exact test for categorical variables. Statistical significance was set at 0.05, and lower *p* values were considered significant. Twelve month follow‐up data were available for all patients. The 12‐month cumulative rates of TLF, MACE, all‐cause death, and TLR in the groups compared by the log‐rank test are shown in the Kaplan–Meier curves.

## 3 Results

### 3.1 Baseline Clinical, Angiographic, and Procedural Characteristics

The DK culotte cohort consisted of 49 patients, while 52 patients were assigned to the culotte arm. There were no significant differences between the two study groups with respect to these characteristics. The mean age of the patients in both groups was similar and reached 67 years, with a predominance of males in each group (67.3% in the DK culotte group and 59.6% in the culotte group) (see Table 1).

**Table 1:** Baseline clinical characteristics of both study cohorts.

	DK Culotte (*n* = 49)	Culotte (*n* = 52)	*p* value
Age, years	67 ± 9.5	67.1 ± 9.4	0.943
Male sex	33 (67.3%)	31 (59.6%)	0.536
Unstable angina	24 (49%)	16 (30.8%)	0.070
NSTEMI	18 (36.7%)	26 (50%)	0.229
STEMI	7 (14.3%)	10 (19.2%)	0.599
*Comorbidities*			
Diabetes mellitus type 2	14 (28.6%)	25 (48.1%)	0.065
Insulin‐dependent diabetes	6 (12.2%)	5 (9.6%)	0.756
Hypertension	40 (81.6%)	42 (80.8%)	1
Hyperlipidemia	41 (83.7%)	36 (69.2%)	0.105
Atrial fibrillation	7 (14.3%)	12 (23.1%)	0.314
Current smoking	20 (40.8%)	15 (28.8%)	0.218
COPD/asthma bronchial	1 (2%)	6 (11.5%)	0.113
Previous PCI	14 (28.6%)	17 (32.7%)	0.673
Previous MI	13 (26.5%)	10 (19.2%)	0.478
Previous CABG	2 (4.1%)	2 (3.8%)	1
Dialysis	1 (2%)	0 (0%)	0.485
Ischemic stroke/TIA history	0 (0%)	5 (9.6%)	0.057
LVEF, %	55 [45–61]	55 [45–60]	0.558
*Laboratory tests*			
Total cholesterol, mmol/L	4.7 ± 1.2	5.1 ± 1.5	0.207
LDL, mmol/L	2.6 [1.9–3.6]	2.9 [2–3.6]	0.360
HDL, mmol/L	1.2 [1.1–1.4]	1.2 [1–1.5]	0.739
Hemoglobin, baseline, g/dL	14.3 [13.6–15.4]	14.1 [13–14.9]	0.323
Creatine, µmol/L	82 [71–94.5]	82.6 [71.3–95.3]	0.895
*Antiplatelets and anticoagulants at discharge*			
ASA	49 (100%)	52 (100%)	
Clopidorel	29 (59.2%)	38 (73.1%)	0.148
Tikagrelor	17 (34.7%)	14 (26.9%)	0.518
Prasugrel	2 (4.1%)	0 (0%)	0.233
NOAC	6 (12.2%)	7 (13.5%)	1
VKA	2 (4.1%)	3 (5.8%)	1

*Note:* Values are *n* (%), mean ± SD or median [interquartile range].

Abbreviations: ASA, acetylsalicylic acid; CABG, coronary artery bypass graft; COPD, chronic obstructive pulmonary disease; DK, double kiss; HDL, high‐density lipoprotein; LDL, low‐density lipoprotein; LVEF, left ventricular ejection fraction; MI, myocardial infarction; N/A, not applicable; NOAC, novel oral anticoagulant; PCI, percutaneous coronary intervention; SD, standard deviation; TIA, transient ischemic attack, VKA, vitamin K antagonists.

There were no significant differences in the anatomic progression of coronary artery disease between the study cohorts, with the mean Syntax score reaching 13 [9, 10, 11, 12, 13, 14, 15, 16, 17, 18, 19, 20] in the DK‐culotte group and 14.5 [10–19.2] in the culotte cohort (*p* = 0.833). There were no significant differences in terms of procedural features. Predominantly operators used radial access 87.8% in DK‐ culotte and 86.5% in the culotte group (see Table 2).

**Table 2:** Baseline angiographic and procedural characteristics of both study cohorts.

	DK Culotte (*n* = 49)	Culotte (*n* = 52)	*p* value
SYNTAX score I	13 [10–21]	14.5 [10–19.2]	0.833
Logistic SYNTAX score	2.9 [1.9–5.1]	3.3 [1.8–10.3]	0.291
PCI SYNTAX score II	31.6 ± 11.7	34.4 ± 15.6	0.307
*Bifurcation localization*			
LM	10 (20.4%)	4 (7.7%)	0.086
Non‐LM (LAD/D)	26 (53.1%)	26 (50%)	0.843
Non‐LM (Cx/OM)	11 (22.4%)	18 (34.6%)	0.194
non‐LM (RCA/PLA)	2 (4.1%)	4 (7.7%)	0.679
*Procedure characteristics*			
Femoral access	6 (12.2%)	7 (13.5%)	1
Radial access	43 (87.8%)	45 (86.5%)	1
Bail out two stent strategy	2 (4.1%)	4 (7.7%)	0.679
Side branch stent diameter, mm	2.5 [2.5–2.8]	2.5 [2.5–2.8]	0.434
Side branch stent length, mm	20 [16–26]	22 [18–28]	0.425
Main branch stent diameter, mm	3 [2.8–3.5]	3 [3]	0.461
Main branch stent length, mm	22 [18–34]	26 [18–33.2]	0.451
Stent to the side branch first	45 (91.8%)	44 (84.6%)	0.356
KB after the first stent implantation	49 (100%)	0 (0%)	< 0.0001
KB after the second stent implantation	49 (100%)	50 (96.2%)	0.495
IVUS/OCT imaging	4 (8.2%)	0 (0%)	0.052
Rotablation	3 (6.1%)	2 (3.8%)	0.672
Intravascular lithotripsy	1 (2%)	0 (0%)	0.485
GP IIb/IIIa use	0 (0%)	4 (7.7%)	0.118
Radiation dose (mGy)	1648 [1166–2465]	1819 [1029–3266.8]	0.497
Contrast media amount (mL)	220 [185–300]	220 [178–260]	0.554

*Note:* Values are *n* (%), mean ± SD, or median [interquartile range].

Abbreviations: Cx, circumflex; D, diagonal; DK, double kiss; GP, glycoprotein; IVUS, intravascular ultrasound; KB, kissing balloon; LAD, left anterior descending; LM, left main; N/A, not applicable; OCT, optical coherence tomography; OM, obtuse marginal; PCI, percutaneous coronary intervention; PLA, posterolateral artery; POT, proximal optimization technique; RCA, right coronary artery; RVA, right ventricular artery; SD, standard deviation.

### 3.2 Clinical Outcomes

**Figure 2:**
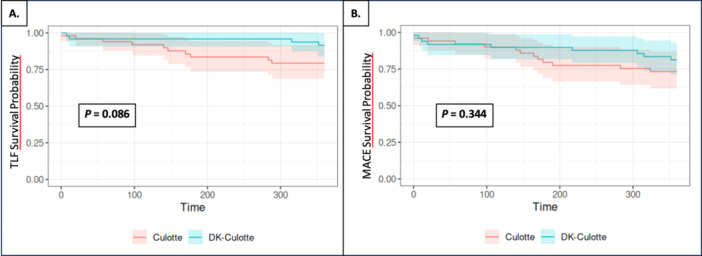
(A) TLF 12 months Survival‐Free Kaplan–Meier curves (B) MACE 12 months Survival‐Free Kaplan–Meier curves.

There were no statistically significant differences in the TLF rate between the two study cohorts at 12 months. However, a trend favoring the DK culotte was observed (8.2% vs. 19.2%, *p* = 0.086). Figure 2 presents Kaplan–Meier curves that demonstrate the TLF and MACE free survival. A comparable consistent trend was observed with regard to TLR (5.13% vs. 9.02%, *p *= 0.162), predominantly due to a reduced incidence of stent restenosis in the DK culotte cohort in comparison to the culotte arm (4.1% vs. 9.6%; *p* = 0.203). A comparison of contrast utilization and radiation dose in both the study cohorts revealed no significant differences in the total amount used during PCI procedures. Similarly, no statistically significant differences were observed in the incidence of other study endpoints, including MACE, target lesion MI, and all‐cause mortality, between the two study cohorts. All data are presented in Table 3.

**Table 3:** Study outcomes.

	DK Culotte (*n* = 49)	Culotte (*n* = 52)	*p* value (Log‐rank)
*12‐month follow‐up primary outcome*			
TLF (cardiac death, TV‐MI, TLR)	4 (8.2%)	10 (19.2%)	0.086
*12‐month follow‐up secondary outcome*			
MACE (MI, cardiac death, TLR)	9 (18.4%)	13 (25%)	0.344
TLR	3 (6.1%)	7 (13.5%)	0.162
All‐cause mortality	4 (8.2%)	5 (9.6%)	0.732
Stent thrombosis	2 (4.1%)	3 (5.8%)	0.660
Stent restenosis	2 (4.1%)	5 (9.6%)	0.203

*Note:* Values are *n* (%).

Abbreviations: MACE, major adverse cardiac events; TLF, target lesion failure; TLR, target lesion revascularization.

## 4 Discussion

The main finding of our study suggests that the DK culotte technique may be associated with a reduction in TLF and MACE compared to the culotte technique, although this difference did not reach statistical significance and should be interpreted with caution.

Coronary bifurcations are a rather complex clinical scenario, and their optimal treatment method continues to be part of substantial debate. A growing number of evidence suggests that provisional stenting is generally recommended [10] for the vast majority of bifurcation lesions, as several randomized and registry studies [11, 12] have shown better or similar long‐term clinical outcomes with single stenting compared to dual stenting techniques. However, some data suggest that this favorable clinical effect is attenuated in a subset of patients with non‐LM bifurcation [13]. It is beyond question that particularly in the ACS population, acute occlusion of a side branch has short‐ and long‐term clinical implications [14]. Despite fact that the importance of vessels below 2.5 mm reference diameter is frequently underestimated in routine clinical practice, they can undoubtedly exert a strong influence on long‐term outcomes [15].

Therefore, maintaining side branch patency during the intervention in the ACS bifurcation is an important factor in determining the initial PCI strategy. Several anatomic factors may potentially predict side‐branch occlusion [16, 17] however only a few of these [9] may suggest clinical benefit from the use of a two‐stent technique as a systematic approach to managing bifurcation lesions.

Several studies suggest that the DK crush technique may have potential advantages over the “classic crush” and other two‐stent techniques for LM bifurcations; however, concordant data are lacking for non‐LM lesions, particularly those involving small side branches. LM bifurcation, in particular, remains a crucial area of focus in bifurcation interventions. In the context of the present subanalysis, the LM subpopulation is scarred, and statistically underpowered. A recently published subanalysis of the LSCBR registry [19] includes a larger cohort of patients treated for LM disease using both the DK culotte and culotte techniques. The results of this analysis revealed no statistically significant difference between the two techniques; however, the DK technique demonstrated a more favorable trend in terms of clinical performance.

Final kissing has been shown to improve the clinical outcome of bifurcation treatment in both 1‐stent and 2‐stent strategies [20, 21, 22]. However, the clinical data regarding the effect of the additional second kissing balloon during the DK culotte is still lacking [6, 7]. Our study, to the best of our knowledge, is the first to focus on this issue. One‐year results of our study showed no significant differences in terms of TLF between both study groups (8.2% vs. 19.2%), however, we could observe a favorable trend in the DK culotte group in terms of TLF (*p* = 0.086). This finding was associated with a lower number of TLR (6.1% vs. 13.5%; *p* = 0.162), probably related to a reduction in stent restenosis (4.1% vs. 9.6%; *p* = 0.203). It is worth noting that additional KB had no impact on procedure safety features (contrast volume, radiation dose). We can only hypothesize that in a longer follow‐up, this trend will gain strength and be similar to results observed in terms of DK crush. However, clinical evidence will be necessary in support of this hypothesis.

Unfortunately, comparable data regarding bifurcation lesions involving small vessels treated with two‐stent techniques during ACS are lacking. It is, therefore, crucial to emphasize that the cohort of the present study is at a high risk of adverse outcomes. Consequently, any comparisons with previously published data must be made with caution. On the one hand, ACS has been described as an independent risk factor for bifurcation lesions [23] On the other hand, PCI in small vessels has been associated with a higher rate of adverse events, particularly concerning bifurcation lesions [24, 25, 26]. Furthermore, the high degree of complexity of the lesions is evidenced by the high average Syntax score in both study arms, reflecting the high number and length of implanted stents. Consequently, any comparisons with previously published data must be made with caution.

Although methodological drawbacks associated with direct comparison are acknowledged, the outcomes observed in the DK culotte group (TLR 6.1%) are promising in comparison to other non‐LM studies. In the available literature, the 1‐year TLR rate in the Culotte group reached up to 6.7% [27, 28] The 1‐year follow‐up TLR after T‐stenting demonstrated values in the range of 2.9%–8.9% [5, 29, 30]. The 1‐year TLF for the classical crush group was found to be in the range of 2.9%–10.5% [31, 32]. Moreover, the outcomes of TLR in the DK‐Culotte arm were nearly identical to those observed in the DK crush stenting group in non‐LM lesions (6.5%) [33], which is currently the preferred two‐stent technique in the latest PCI guidelines.

Despite the unfavorable clinical and anatomic presentation of both study cohorts, the vast majority of procedures were performed via radial access. It can be stated that neither the ACS subset nor the procedural initial complexity inherent to the two‐stent techniques used in the study necessitated conversion to femoral access. This fact may have an effect on the results obtained, in particular with regard to the safety outcomes, due to the strong association between this access point and safety that has been demonstrated previously [34]. Another interesting aspect of this study is that in the baseline characteristics of both groups, the predominance of men (DK culotte: 67.3% and culotte: 59.6%) was not as significant. This likely reflects the fact that interventions involving small vessels more frequently concern women.

## 5 Limitations

Our study meets several limitations, the majority of which are inherent to the retrospective observational study design. The study may lack sufficient statistical power due to the relatively small number of subjects involved. Furthermore, our study was lacking in external core lab evaluation, particularly in the case of quantitative coronary angiography. This may have affected the precision of our findings regarding the characteristics of bifurcation lesions. One of the main limitations of our study is the low utilization of IVUS/OCT imaging, which is known to improve clinical outcomes by reducing adverse events.

## 6 Conclusion

In the context of small vessel stenting, the DK culotte technique may be associated with favorable outcomes in terms of safety and efficacy in the ACS cohort. In our study cohort, the DK culotte technique was associated with a reduction in TLF (8.2% vs. 19.2%, *p* = 0.082) and MACE (18.4% vs. 25%, *p* = 0.344) compared to the culotte technique. However, due to the limited sample size and lack of statistical significance, these observations should be considered preliminary and warrant further investigation.

## Data Availability

The data that support the findings of this study are available on request from the corresponding author. The data are not publicly available due to privacy or ethical restrictions.
